# Anti-vibration method for sensing ring of fiber optical current transformer integrating structural design and adaptive signal processing

**DOI:** 10.1371/journal.pone.0341890

**Published:** 2026-02-27

**Authors:** Lin Cheng, Jianyong Luo, Weibin Si, Yanhua Han, Kun Zuo, Haitao Sun, Bo Niu, Shuangzan Ren

**Affiliations:** 1 State Grid Shaanxi Electric Power Co., Ltd. Electric Power Research Institute, Xi’an, China; 2 State Grid Shaanxi Electric Power Co., Ltd., Xi’an, China; 3 State Grid Shaanxi Electric Power Co., Ltd. Ultra-high Voltage Company, Xi’an, China; University of Baghdad, IRAQ

## Abstract

This paper presents an in-depth study on vibration resistance improvement and fault identification technology for fiber-optic current transformers (FOCTs). The research analyzes the working principle of FOCTs, focusing on the Faraday magneto-optical effect and Ampere’s circuital law, alongside optoelectronic device models. The impact of vibration on FOCT performance is thoroughly investigated through theoretical analysis and experimental research, clarifying how vibration affects components like polarization-maintaining delay fiber and sensing fiber. Experimental studies on random and impact vibration characteristics summarize the variation patterns of transformer performance before and after vibration. To enhance vibration resistance and fault identification, corresponding measures are proposed using ANN-based and FIR filtering-based methods. These methods compensate for measurement errors under sinusoidal (semi-sinusoidal) and impact vibration conditions. The results demonstrate the effectiveness of these techniques in maintaining accurate current measurements despite vibrational interference. The findings provide a theoretical foundation and technical support for improving the operational stability of FOCTs in practical applications, particularly in harsh environments. This combined approach of optimized structural design and adaptive signal processing contributes to the advancement of FOCT technology and its wider adoption in demanding settings.

## 1 Introduction

Fiber optic current transformers (FOCTs) offer significant advantages such as a large dynamic range, high measurement accuracy, immunity to electromagnetic interference, electrical insulation, compact structure, light weight, and excellent transient characteristics [1–3]. They are widely used in ultra-high voltage (UHV) DC converter stations, primarily for measuring DC current and transmitting the results to DC control and protection equipment [4]. UHV technology, as a major original innovation developed in China with world-leading capabilities and independent intellectual property rights, has solved the global challenge of long-distance, large-capacity, low-loss power transmission. It is the core technology for constructing large-scale interconnected power grids and achieving efficient, optimized allocation of clean energy nationwide.

The fiber-optic current transformer is an optical interferometric instrument with a complex internal structure that composed of numerous optical and electronic components, which fully utilizes the anti-interference effect of optical fibers [5]. During actual operation, FOCTs are susceptible to various external factors (e.g., temperature, vibration interference), leading to decreased measurement accuracy or even failure [6,7]. To enhance the operational stability of FOCTs within converter stations, it is imperative to deeply investigate the mechanisms by which vibration affects FOCTs, propose measures to improve vibration resistance, and ensure the safe and stable operation of DC measurement equipment within the stations. Especially, FOCTs face extensive vibration environmental impacts, posing great challenges to the high reliability application of sensors [8,9].

Regarding FOCT vibration resistance enhancement and fault identification technology, numerous research efforts have been conducted by scholars domestically and internationally. For instance, Rogers et al. modified the fiber optic gyroscope scheme, resulting in a closed-loop optical path between the phase modulator and the sensing fiber ring, exhibiting weak vibration resistance [10]. Short et al. proposed a linear FOCT scheme by introducing a straight waveguide phase modulator supporting birefringence modulation, which significantly suppressed the influence of the Sagnac effect on the optical path [11]. Subsequent improvements and optimization strategies have largely been based on this optical structure. For example, Bohnert et al. investigated the immunity of different FOCT structures to vibration interference, finding that a non-reciprocal sensing optical path with ring winding is more sensitive to external vibration interference than a reciprocal reflective sensing optical path structure [12]. Jiang et al. were the first to study the impact of acoustic vibration on FOCTs, concluding that the vibration resistance of inline interferometric FOCTs is significantly superior to that of polarization-based FOCT schemes [13]. Wang et al. proposed an FOCT scheme insensitive to both temperature and vibration [14], in which a reciprocal optical path structure with an inline straight waveguide phase modulator is employed to enhance optical path vibration resistance, and complex sensing fiber encapsulation processes is utilized to reduce mechanical stress on the fiber. Yuan et. al indicates that research on digitally closed-loop phase-modulated FOCTs was initiated based on advancements in domestic digitally closed-loop fiber optic gyroscope technology and mastery of lithium niobate phase modulator manufacturing [15]. Zhao et. al proposed an FOCT scheme to eliminate vibration sensitivity and temperature drift. It employed a double-wire winding method for the fiber coil, achieving good system linearity with relative deviations not exceeding ±0.2% [16]. Temperature drift was essentially eliminated within −50 °C to 50 °C range. This transformer features a simple structure, high precision, and strong anti-interference capability, offering new ideas for FOCT design. However, this scheme only effectively suppresses low-frequency vibration and still has a considerable gap to practical engineering applications. Mu et. al proposed a vibration-immune fiber sensing ring winding scheme [17]. However, quantitative results was not provided in this study on vibration interference suppression nor developed corresponding engineering products. Li et. al proposed a novel high-birefringence spun fiber winding method to suppress the temperature and vibration sensitivity of the sensing ring [18]. Kang et. al conducted theoretical research on the error characteristics of marine FOCTs under vibration and impact, identifying the polarization-maintaining fiber delay loop as the primary cause of vibration errors [19]. However, since the vibration impact in shipboard environments mainly originates underwater, findings of this work are not applicable to other application scenarios. Wang et. al proposed an FOCT capable of simultaneously measuring current and vibration [20], which is only suitable when the frequency characteristics of the measured current signal and the vibration interference signal do not overlap. Kim et al conducted evaluation of the structural vibration effect on Plasma current measurement using the fiber optic current sensor in International Thermonuclear Experimental Reactor (ITER) project, and results show that the vibration effect can be strongly reduced when a proper low-birefringent spun optical fiber is used [21]. Xiang et al proposed a novel polarimetric fiber optic current sensor based on orbital angular momentum modes of an air-core optical fiber, which effectively measures polarization rotation at the output of a fiber in a magnetic field [22].

In summary, worldwide research on the vibration resistance characteristics of FOCTs mostly remains at the laboratory validation stage, focusing primarily on improving the vibration resistance of either the fiber sensing ring or the optical path acquisition module. Due to a lack of practical engineering experience, there is a scarcity of research findings reported on the vibration interference characteristics of the polarization-maintaining transmission tail fibers associated with the sensing ring. Therefore, it is urgent to conduct comprehensive and in-depth research into the mechanisms of FOCT vibration errors to resolve the overall vibration interference error problem in FOCTs. In this work, we proposed a novel method for sensing ring combining structural design and adaptive signal process. A special design of fiber optic current sensing ring structure is designed for anti-vibration. Sinusoidal and semi-sinusoidal vibration experiments were conducted to verify that the designed structure exhibits high-precision current measurement under sensing ring vibration measurement.

Rest of this paper are organized as follows. Section 2 introduces the principle of fiber-optic current transformer. Section 3 conduct theoretical analysis of vibration error of fiber-optic current transformer. Section 4 introduces the experimental system and results of current measurement under semi-sinusoidal vibration and sinusoidal vibration. Section 5 introduces the experimental system and results of current measurement under optical cable impact. Section 6 proposes current signal noise suppression method optimized threshold wavelet. Finally, main findings and conclusions are drawn in Section 7.

## 2 Principle analysis of fiber-optic current transformer

The fiber-optic current transformer functions current measurement based on the Faraday magneto-optical effect and Ampere’s circuital law. The polarization plane of linearly polarized light rotates when passing through a magneto-optical crystal. The rotation angle is related to factors such as the Verdet constant of the crystal and the magnetic field strength. The magnetic field is linked to the measured current via Ampere’s circuital law, thereby enabling current measurement. The optical structure includes components such as a light source, polarizer, phase modulator, and photodetector [23].

FOCT Optoelectronic Device Models is introduced as follows:

(1) SLD light source: The Super luminescent Diode (SLD) features high output optical power, broad spectral width, short temporal coherence, and long spatial coherence [24]. It is the commonly used semiconductor light source for FOCTs. Its internal components include the SLD chip, Thermoelectric Cooler (TEC), and thermistor. It typically emits partially polarized light.
(2) Fiber polarizer: Polarizers with high extinction ratios are crucial for suppressing polarization errors in transformers for polarizing and analyzing polarization [25]. Common types include mode attenuation and mode cutoff types.
(3) Optical phase modulator: This core device introduces non-reciprocal modulation phase into the FOCT’s optical system [26]. Open-loop control FOCTs often use piezoelectric ceramic materials (Lead Zirconate Titanate, PZT) as the main modulation element. Lithium Niobate (LiNbO3) phase modulators are commonly used in closed-loop control FOCTs, based on the material’s Pockels electro-optic effect.
(4) Polarization-maintaining (PM) Delay Loop: The PM delay loop serves as the light transmission medium between the high-voltage side sensing fiber ring and the low-voltage side signal processing unit [27]. By applying stress on both sides of the fiber core or creating a non-circular geometric core shape, two well-defined birefringent principal axes (slow axis and fast axis) are formed, maintaining the polarization state of the transmitted light wave.
(5) Fiber *λ/4* waveplate: The fiber *λ/4* waveplate utilizes stress or shape birefringence between the two polarization axes of PM fiber, inducing a phase difference between the orthogonal components propagating along the fast and slow axes, thereby altering the light’s polarization state [28]. Its polarization coupling model is influenced by the actual manufacturing process and temperature environment.
(6) Fiber fusion point: Axial alignment errors exist between the PM tail fibers of various components during the fiber fusion splicing process, manifested as a polarization coupling point characterized by the misalignment angle.
(7) Sensing fiber ring: A closed loop formed by sensing fiber with a mirror at its end, used to measure the Faraday phase shift generated by the conductor current [29]. The sensing fiber ring maintains stable transmission of the circular polarization state, enabling the transformer to accurately acquire the Faraday phase shift. Its model considers factors such as linear birefringence, circular birefringence, and the Faraday phase shift.
(8) PIN-FET photodetector: A photoelectric conversion device offering good linearity, high sensitivity, low noise, fast response speed, high temperature stability, and no requirement for high operating voltage [30]. The component consists of a PIN photodiode, FET preamplifier circuit, and tail fiber assembly, converting optical signals into electrical signals.

Open-loop signal detection principle: In open-loop FOCTs, the sinusoidal modulation scheme is employed, the Faraday phase shift angle within the transformer is demodulated by analyzing the output interference signal from the photodetector, employing methods like Bessel function expansion [31], thereby achieving current measurement. Simultaneously, to improve modulation depth stability, feedback compensation utilizes the amplitudes of the second and fourth harmonic components at the modulation frequency within the output signal.

Closed-loop signal detection principle: FOCTs achieve high-precision, large-dynamic-range signal detection by using all-digital phase modulation/demodulation technology and closed-loop feedback techniques [32]. Methods such as square-wave modulation, digital correlation detection, and staircase-wave feedback ensure stable closed-loop system output, enabling accurate measurement of the current under test. The mathematical model of the closed-loop detection system can be further analyzed for system stability and measurement accuracy characteristics.

## 3 Vibration error analysis of fiber-optic current transformer

### 3.1 Analysis of vibration impact on polarization-maintaining delay fiber

It is assumed the stress wave applied to a micro-segment of the delay fiber follows a sinusoidal pattern with frequency *ω* and induced phase shift amplitude *φ*_*0*_*.* The time-dependent phase shift due to stress is:


$$\varphi \left(t\right)=\varphi_{\text{0}}\;sin\left(\omega t\right)$$ (1)

At time *t*_1_, the fiber segment with the length of *∆l* is considered located at distance *l* from the reflector. Under the aforementioned vibrational stress, the phase difference generated between the two polarized beams is:


$$\Delta \varphi \left(t_{\text{1}}\right)=K\Delta l\left(2\;sin\left(\omega t_{\text{1}}\right){sin}^{\text{2}}\left(\frac{\omega nl}{c}\right)+cos\left(\omega t_{\text{1}}\right)sin\left(\frac{2\omega nl}{c}\right)\right)$$ (2)

where *K* denotes the calculating coefficient, Δ*l* denotes the length of the fiber segment, *l* denotes the distance between the fiber and the reflector, *c* denotes the speed of light, *n* denotes the refractive index.

Considering vibration frequencies below 1kHz and the short delay parameter of the FOCT (microsecond level), the second-order small quantities in the above equation can be neglected, simplifying it to:


$$\Delta \varphi \left(t_{\text{1}}\right)=K\;cos\left(\omega t_{\text{1}}\right)sin\left(\frac{2\omega nl}{c}\right)\Delta l$$ (3)

It is assumed that the total length of the delay fiber loop is *L*, and the distance from its start to the reflector is *l*_1_. The overall non-reciprocal phase angle induced in the delay fiber loop under vibration is obtained by integrating the above expressions, which can be expressed as:


$$\Delta \varphi_{r}\left(t_{\text{1}}\right)=K\Delta l^{\text{'}}cos\left(\frac{2\omega nl_{\text{1}}+2\omega nL}{c}\right)-K^{\text{'}}cos\left(\frac{2\omega nl_{\text{1}}}{c}\right)$$ (4)

**Fig 1:**
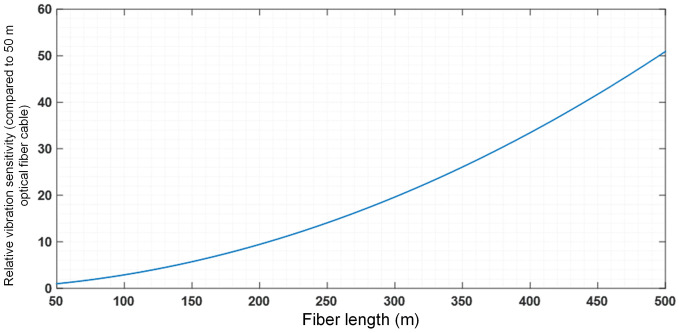
Relative vibration sensitivity of transformer on delay fiber.

Further, the vibration frequency is set to 1000 Hz, with a refractive index of *n* = 1.46 (refractive index of fiber’s core), *l*_1_ = 30 meters, *L* ranging from 50 to 500 meters, and the non-reciprocal phase angle for different *L* values is normalized relative to *L* = 50 meters. The simulation result of the above equations is shown in Fig 1.

It is evident that shortening the length of the delay fiber coil can effectively reduce sensitivity to vibration. It is considered that the modulation frequency of PZT modulators is around 100 kHz. Hence, the delay fiber coils are typically determined as 500 m, which makes the fiber significantly affected by vibration. If the phase modulator is replaced with a straight waveguide electro-optic modulator, the modulation frequency can be increased to 1 MHz, allowing the delay fiber loop length to be shortened to 50 m. This reduces vibration sensitivity to approximately 2%.

### 3.2 Analysis of vibration impact on sensing fiber

The intrinsic linear birefringence in optical fiber can be expressed as:


$$\beta_{g}=\beta_{f}+\beta_{y}$$ (5)

where *β*_*f*_ denotes the linear birefringence caused by core non-circularity, *β*_*y*_ denotes the linear birefringence caused by residual stress in the fiber.

Since the core cross-section becomes elliptical during manufacturing under various combined influences [33,34], the linear birefringence caused by core non-circularity arises. The major axis of the elliptical core cross-section is as the valuable of *a*. The two orthogonal polarized beams propagate along the major and minor axes of the ellipse, respectively. The expression for the phase difference generated between them is:


$$\beta_{f}=\frac{e^{\text{2}}}{8a}{\left(2\Delta n\right)}^{\frac{\text{3}}{\text{2}}}f\left(V\right)$$ (6)

$$V=\frac{2\pi a}{\lambda }N_{a}$$ (7)

where *e* represents the core ellipticity, *∆n* represents the relative refractive index difference, *V* is the normalized frequency, *a* represents the long semiaxis of ellipse, *N*_*a*_ represent the numerical aperture, *λ* represents the wavelength in vacuum.

Linear birefringence due to residual stress in optical fiber arises from inconsistent thermal expansion coefficients between the core and cladding materials, creating internal stress imbalance and inducing anisotropy in the core material [35]. The expression is:


$$\beta_{y}=\frac{\pi n^{\text{3}}}{\lambda E}\left(1+\rho \right)\left(p_{12}-p_{11}\right)\sigma$$ (8)

where *n* represents the fiber refractive index, *λ* is the optical wavelength, *E* represents Young’s modulus, *ρ* is Poisson’s ratio, *p*_12_ and *p*_11_ represent the elasto-optic coefficients, and *σ* represents the stress difference between two orthogonal directions in the core.

Linear birefringence induced by external factors is more complex, primarily caused by anisotropy in the fiber material resulting from bending, twisting, vibration, etc., under external forces during operation [36]. For FOCTs, since the sensing fiber forms a closed ring structure, the optical fiber bending is inevitable, leading primarily bend-induced linear birefringence in the sensing fiber. The expression for bend-induced linear birefringence in sensing fiber is:


$$\beta_{w}=\frac{\pi n^{\text{3}}}{2\lambda }\left(1+\rho \right)\left(p_{12}-p_{11}\right){\left(\frac{A}{R}\right)}^{\text{2}}$$ (9)

where *A* denotes the outer diameter of the sensing fiber, *R* represents the bending radius of the sensing fiber.

Additionally, bending the sensing fiber also alters the cross-sectional shape of the core, transforming it from circular to elliptical. The resulting ellipticity is:


$$\beta_{f}^{*}=\frac{\pi \rho^{\text{2}}a{\left(2\Delta n\right)}^{\frac{\text{3}}{\text{2}}}}{4R}$$ (10)

Based on the above analysis, linear birefringence always exists in the sensing fiber. The Jones matrix of the sensing ring can be expressed as:


$$M_{Fin}=M_{Fout}=\left[\begin{array}{cc} @cc@A & -B\\ B & A^{*} \end{array}\right]$$ (11)

$$\left\{ \begin{array}{c} @c@\begin{array}{c} A=cos\left(\frac{\theta }{\text{2}}\right)+j\;sin\left(\frac{\theta }{\text{2}}\right)cos\;\varphi \\ B=sin\left(\frac{\theta }{\text{2}}\right)sin\;\varphi \end{array}\\ A^{\text{*}}=cos\left(\frac{\theta }{\text{2}}\right)-j\;sin\left(\frac{\theta }{\text{2}}\right)cos\;\varphi \\ \theta =\sqrt{4F^{\text{2}}+\beta^{\text{2}}}\\ tan\;\varphi =\frac{2F}{\beta } \end{array}\right.$$ (12)

where *F* represents the Faraday phase shift angle in the sensing fiber *F = VNI*, *V* represents the Verdet constant, *N* represents the number of sensing fiber turns, *I* represents the measured current, and *β* represents the total linear birefringence in the sensing fiber.

Therefore, when linear birefringence is present in the sensing fiber, the expression for the output interference light intensity of the FOCT is:


$$I_{out}^{*}=\frac{\text{1}}{\text{4}}I_{\text{0}}\left[1+\left(1-\frac{2F}{\sqrt{\beta^{\text{2}}+4F^{\text{2}}}}\right)cos\;\varphi_{b}+\frac{2F}{\sqrt{\beta^{\text{2}}+4F^{\text{2}}}}\;cos\left(2\sqrt{\beta^{\text{2}}+4F^{\text{2}}}-\varphi_{b}\right)\right]$$ (13)

where *I*_0_ represents the light source output intensity, *φ*_*b*_ is the bias modulation phase difference. The square-wave modulation is employed, with *φ*_*b*_ value of ±π/2, hence:


$${I_{out}^{*}|}_{\varphi_{b}=\frac{\pi }{\text{2}}}=\frac{\text{1}}{\text{4}}I_{\text{0}}\left[1+\frac{2F}{\sqrt{\beta^{\text{2}}+4F^{\text{2}}}}sin\left(2\sqrt{\beta^{\text{2}}+4F^{\text{2}}}\right)\right]$$ (14)

$${I_{out}^{*}|}_{\varphi_{b}=-\frac{\pi }{\text{2}}}=\frac{\text{1}}{\text{4}}I_{\text{0}}\left[1-\frac{2F}{\sqrt{\beta^{\text{2}}+4F^{\text{2}}}}sin\left(2\sqrt{\beta^{\text{2}}+4F^{\text{2}}}\right)\right]$$ (15)

Since *F*^*2*^≪ *β*^*2*^, the above expressions can be approximated expressed as:


$$\Delta_{I_{out}^{*}}=I_{\text{0}}NV\frac{sin\;\text{2}\beta }{\beta }I$$ (16)

where *N* represents the number of sensing ring turns, *V* represents the Verdet constant of the sensing fiber (typically 1.1 × 10 ⁻ ⁶ rad/A), *I* represents the measured current. Based on this equation, the relative measurement error of the FOCT in the presence of linear birefringence in the sensing fiber can be derived:


$$\varepsilon_{\beta }=\frac{sin\;2\beta }{2\beta }-1$$ (17)

**Fig 2:**
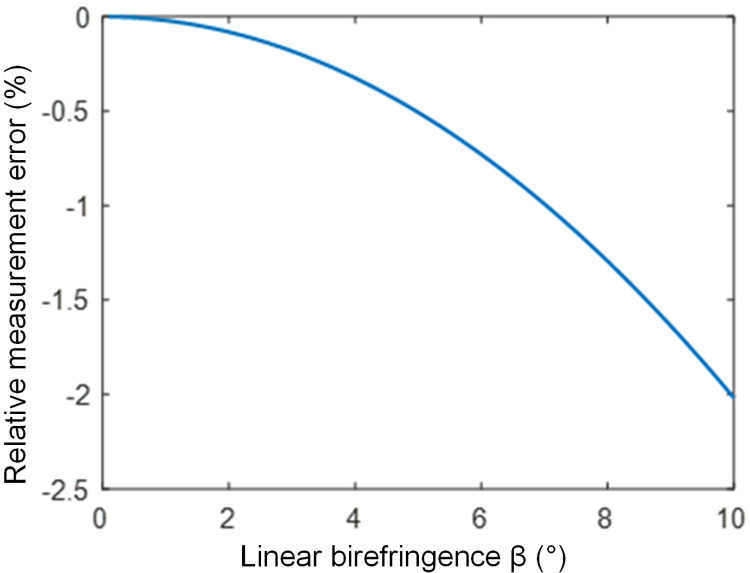
Relationship between FOCT relative error and linear birefringence.

Simulation based on this equation yields the relationship between the relative error of the FOCT current measurement and the linear birefringence in the sensing fiber, as shown in Fig 2.

It can be seen from Fig 2 that linear birefringence in the sensing fiber significantly impacts FOCT measurement accuracy. Based on previous analysis, intrinsic linear birefringence mainly arises from core non-circularity and residual stress. Therefore, to reduce intrinsic linear birefringence, two factors must be controlled during fiber manufacturing: first, improve the sensing fiber manufacturing process to reduce core non-circularity; second, select core and cladding materials with closely matched thermal expansion coefficients to reduce residual stress. Eq. (2) indicates that producing low-birefringence fiber with minimal linear birefringence requires controlling core non-circularity within a very small range, imposing high demands on the manufacturing process. This often results in complex processes, low yield, and high cost. While theoretically feasible, reducing linear birefringence by selecting materials with matching thermal expansion coefficients is practically difficult because core and cladding materials are inherently different [37]. Alternatively, the annealing method can eliminate residual stress in the sensing fiber to reduce linear birefringence. Although this method can substantially decrease linear birefringence, annealed and unannealed fibers are difficult to splice together, posing significant challenges for connecting the optical path system of FOCTs.

Another method to mitigate the impact of linear birefringence on FOCT measurement accuracy is to introduce a certain amount of circular birefringence into the fiber sensing ring [38]. Since the Faraday effect is essentially magnetically induced circular birefringence, introducing circular birefringence allows it to interact additively with the Faraday effect, reducing the influence of linear birefringence and improving FOCT detection accuracy. Furthermore, because the introduced circular birefringence is reciprocal, the phase shift it causes when light passes forward and backward through the sensing fiber cancels out. Therefore, the externally introduced circular birefringence does not affect the transformer’s measurement accuracy. When both linear and circular birefringence coexist in the sensing fiber, the Jones matrix for light propagating forward through the sensing ring is:


$$M_{Hin}=\left[\begin{array}{cc} @cc@C & -D\\ D & C^{*} \end{array}\right]$$ (18)

where:


$$\left\{ \begin{array}{c} @c@\begin{array}{c} C=cos\left(\frac{\theta }{\text{2}}\right)+j\;sin\left(\frac{\theta }{\text{2}}\right)cos\;\varphi \\ D=sin\left(\frac{\theta }{\text{2}}\right)sin\;\varphi \end{array}\\ C^{\text{*}}=cos\left(\frac{\theta }{\text{2}}\right)-j\;sin\left(\frac{\theta }{\text{2}}\right)cos\;\varphi \\ \theta =\sqrt{4{\left(F+\Gamma \right)}^{\text{2}}+\beta^{\text{2}}}\\ tan\;\varphi =\frac{2\left(F+\Gamma \right)}{\beta } \end{array}\right.$$ (19)

where *Γ* is the introduced circular birefringence. After reflection by the mirror, the light propagates backward through the sensing fiber. The Jones matrix for backward propagation is:


$$M_{Hin}=\left[\begin{array}{cc} @cc@C & -D\\ D & C^{*} \end{array}\right]$$ (20)

where:


$$\left\{ \begin{array}{c} @c@\begin{array}{c} C=cos\left(\frac{\theta }{\text{2}}\right)+j\;sin\left(\frac{\theta }{\text{2}}\right)cos\;\varphi \\ D=sin\left(\frac{\theta }{\text{2}}\right)sin\;\varphi \end{array}\\ C^{\text{*}}=cos\left(\frac{\theta }{\text{2}}\right)-j\;sin\left(\frac{\theta }{\text{2}}\right)cos\;\varphi \\ \theta =\sqrt{4{\left(F-\Gamma \right)}^{\text{2}}+\beta^{\text{2}}}\\ tan\;\varphi =\frac{2\left(F-\Gamma \right)}{\beta } \end{array}\right.$$ (21)

When the introduced circular birefringence in the sensing fiber is much greater than the linear birefringence, the Jones matrix of the sensing ring can be approximated as:


$$M_{Hin}=\left[\begin{array}{cc} @cc@cos\left(F+\Gamma \right) & -sin\left(F+\Gamma \right)\\ sin\left(F+\Gamma \right) & cos\left(F+\Gamma \right) \end{array}\right]$$ (22)

$$M_{Hout}=\left[\begin{array}{cc} @cc@cos\left(F-\Gamma \right) & -sin\left(F-\Gamma \right)\\ sin\left(F-\Gamma \right) & cos\left(F-\Gamma \right) \end{array}\right]$$ (23)

The expression for the FOCT output light intensity then can be expressed as:


$$\begin{array}{c} I_{out}^{*}=\frac{\text{1}}{\text{4}}I_{\text{0}}\left\{1+cos\left[2\left(F+\Gamma \right)+2\left(F-\Gamma \right)\right]-\varphi_{b}\right\}\mathrm{\backslash hfill}\\ =\frac{\text{1}}{\text{4}}I_{\text{0}}\left[1+cos\left(4F-\varphi_{b}\right)\right]\mathrm{\backslash hfill} \end{array}$$ (24)

The square-wave modulation is employed, with a *φ*_*b*_ of ±π/2, hence:


$${I_{out}^{*}|}_{\varphi_{b}=\frac{\pi }{\text{2}}}=\frac{\text{1}}{\text{4}}I_{\text{0}}\left[1+sin\left(4VNI\right)\right]$$ (25)

$${I_{out}^{*}|}_{\varphi_{b}=-\frac{\pi }{\text{2}}}=\frac{\text{1}}{\text{4}}I_{\text{0}}\left[1-sin\left(4VNI\right)\right]$$ (26)

Since the FOCT employs a closed-loop signal detection method, the Faraday phase shift within each feedback cycle is very small. The value of *sin(4VNI)* can be approximated as 4*VNI*. Therefore, the equation can be approximated as:


$$\Delta_{I_{out}^{*}}=\text{2}I_{\text{0}}VNI$$ (27)

This indicates that after introducing a certain amount of circular birefringence into the sensing fiber, the output signal of the FOCT approximates the ideal linear model. Circular birefringence can effectively suppress the influence of linear birefringence.

When the sensing fiber is subjected to external vibration or impact, additional linear birefringence is introduced due to the elasto-optic effect [39]. However, when high-birefringence spun fiber is used as the sensing fiber, the inherent circular birefringence within the fiber itself effectively suppresses linear birefringence. Moreover, the left-handed and right-handed circularly polarized light propagating in the sensing fiber can be decomposed into linearly polarized light components with the same orientation. Therefore, when subjected to external vibration or impact, the two interfering light wave signals inherently cancel out most of the error.

## 4 Influence of optical cable impact on the measurement performance of optical fiber current sensors

### 4.1 Experimental system

**Fig 3:**
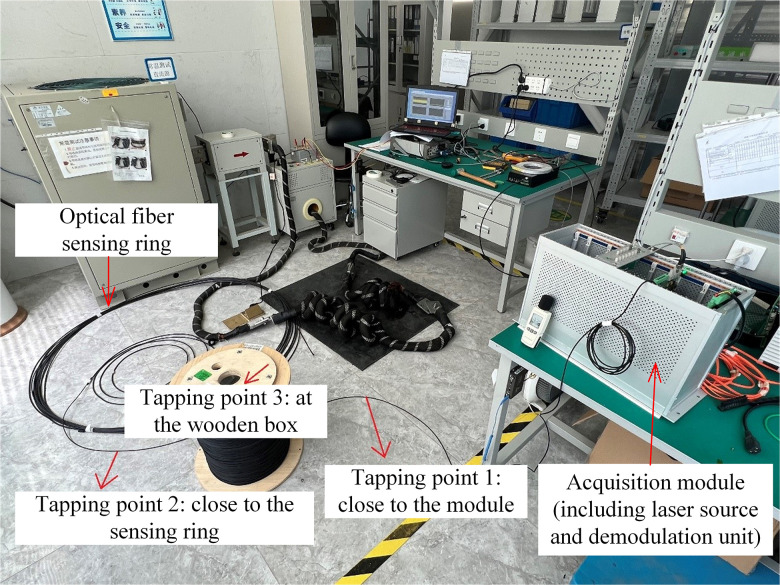
Experimental system for optical cable impact.

Fig 3 shows the physical diagram of the optical cable impact experimental system. Three different tapping point are designed to study the effect of impact location on the measurement performance of optical fiber current sensor, including the tapping point close to the sensing ring, the tapping point close to the module, and the tapping point at the wooden box. In the acquisition module, both laser source and demodulation unit are included. And the detection unit – optical fiber sensing ring is employed with flexible optical fiber rings. In this section, the measured current is the direct current generated by the high current generator, and the standard current is obtained through a standard current transformer. Fiber at each tapping position is tapped multiple times and current measurements were repeated four times.

### 4.2 Experimental results and discussions

**Fig 4:**
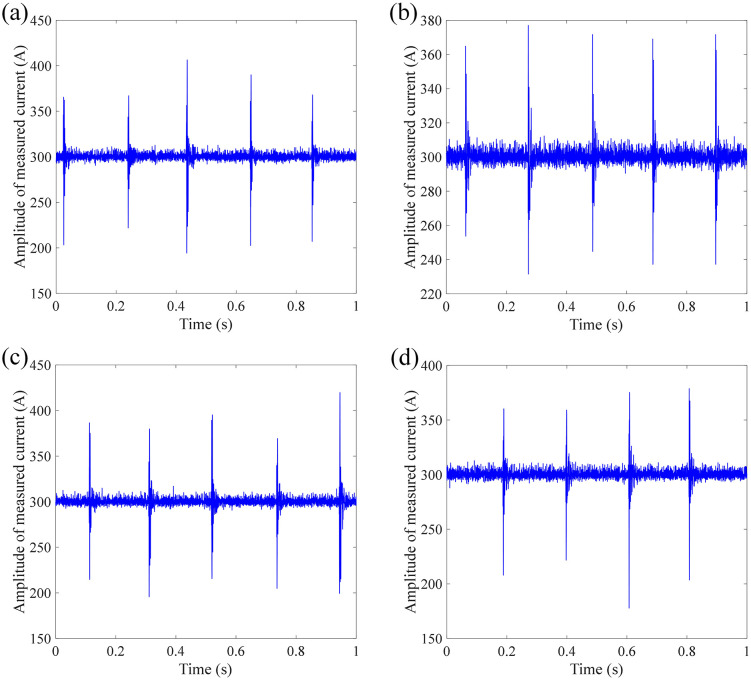
Measurement results of DC current with multiple impacts at position 1: (a) 1^st^ measurement; (b) 2^nd^ measurement; (c) 3^rd^ measurement; (d) 4^th^ measurement.

Fig 4 shows the measurement results of DC current under impact at position 1 (close to the demodulation module). It can be seen from Fig 4 that the current waveform shows several obvious peaks for these four measurements, which correspond to the number and the time of taps applied. In addition to the current spike at the time of the tap, the current signals corresponding to other times when the tap is not received also contain a lot of noise, causing the current to fluctuate around the target current. Hence, the impact makes the FOCT difficult for outputting high-precision current waveform. The RMS between measured current signal and target current signal is calculated as 8.3848 A, 6.5151 A, 9.3458 A, and 6.9714 A, respectively.

**Fig 5:**
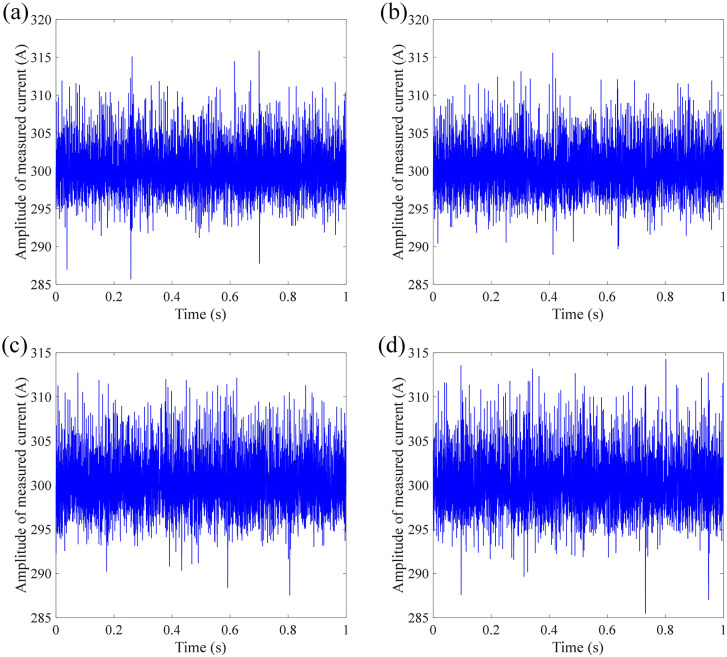
Measurement results of DC current with multiple impacts at position 2: (a) 1^st^ measurement; (b) 2^nd^ measurement; (c) 3^rd^ measurement; (d) 4^th^ measurement.

Fig 5 shows the measurement results of DC current under impact at position 2 (close to the sensing ring). It can be seen from Fig 5 that, although the current fluctuation amplitude is smaller than that when the impact is applied to position 1, the impact applied to the sensing ring will also cause the current signal to contain more noise components, which appears as high-frequency chaotic fluctuations rather than obvious noise peaks. The RMS between measured current signal and target current signal is calculated as 3.2019 A, 3.2262 A, 3.1722 A, and 3.2512 A, respectively. Although the amplitude of noise peak is lower than that at position 1, it is hard to recover the target amplitude from the measured current signal when the shock vibration was applied at position 2.

**Fig 6:**
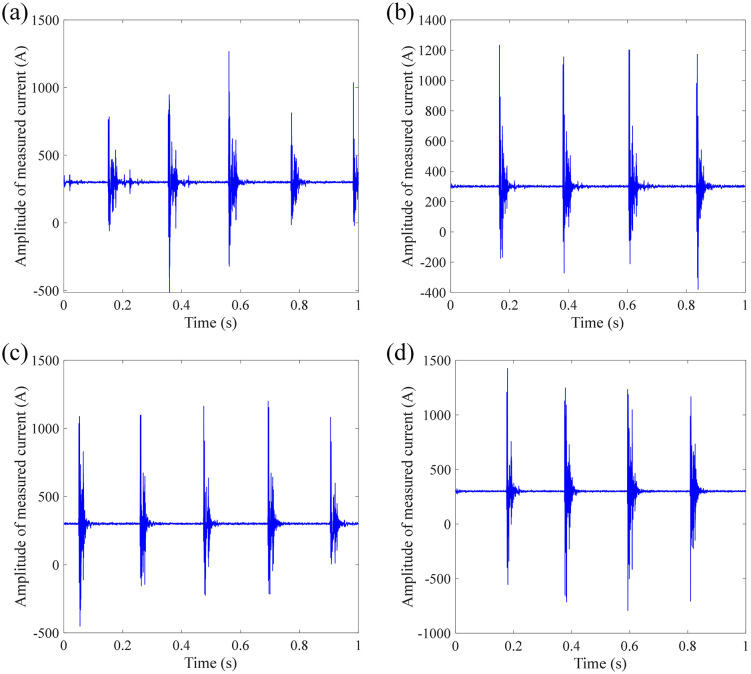
Measurement results of DC current with multiple impacts at position 3: (a) 1^st^ measurement; (b) 2^nd^ measurement; (c) 3^rd^ measurement; (d) 4^th^ measurement.

Fig 6 shows the measurement results of DC current under impact at position 3 (at the wooden box). It can be seen from Fig 6 that, the current waveform is similar with the than that when the impact is applied to position 1, that is, several noise peaks that corresponding with tapping impact are observed in the measured signal. The amplitude of noise peak is higher than that when the impact is applied to position 1. The RMS between measured current signal and target current signal is calculated as 68.8136 A, 105.7062 A, 104.4996 A, and 83.6395 A, respectively.

This section investigated the effect of periodic impact on fiber-optic current transformers. The noise characteristics of FOCTs under periodic impact were experimentally studied. Results show that periodic impact increases the RMS value of the noise data, leading to abrupt points to appear in the noise data. Both the number and amplitude of these abrupt points increase with the impact level.

## 5 Influence of semi-sinusoidal vibration and sinusoidal vibration on the measurement performance of optical fiber current sensors

### 5.1 Experimental system

**Fig 7:**
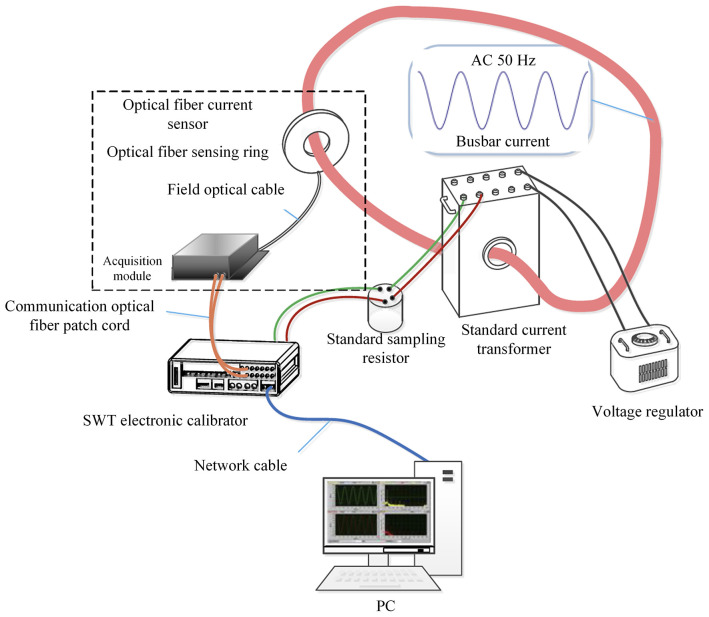
Schematic diagram of fiber sensing ring vibration experiment.

**Fig 8:**
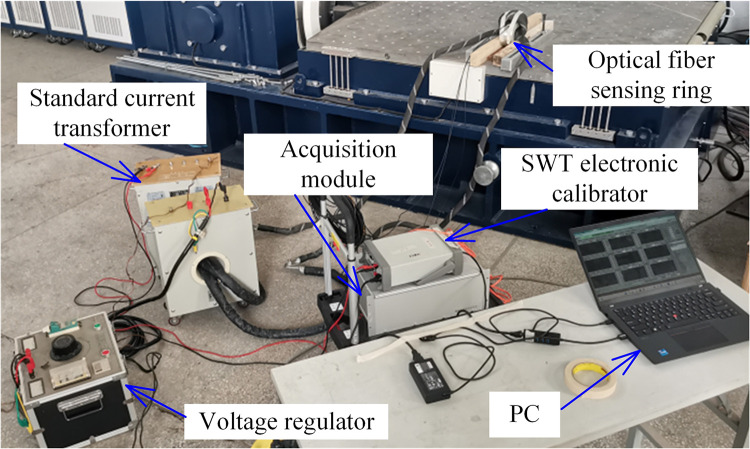
Physical diagram of fiber sensing ring vibration experiment.

The vibration characteristics of the fiber-optic current transformer were studied according to GB/T 2423.11−1997 (Environmental testing for electric and electronic products – Part 2: Test methods – Test Fd: Broadband random vibration – General requirements). Schematic and physical diagram of fiber sensing ring vibration experiment is shown in Figs 7 and 8, respectively. Three different frequencies and two different vibration intensity amplitudes of sinusoidal vibration interference were respectively applied to the optical fiber sensing ring. The output signals of the optical fiber current sensor and the standard transformer were measured, and the output current ratio difference and phase difference of the optical fiber current sensor were recorded. Three different durations and two different intensities of semi-sinusoidal waveform impact interferences were respectively applied to the optical fiber sensing ring. The output signals of the optical fiber current sensor and the standard transformer were measured, and the output current ratio difference and phase difference of the optical fiber current sensor were recorded.

**Fig 9:**
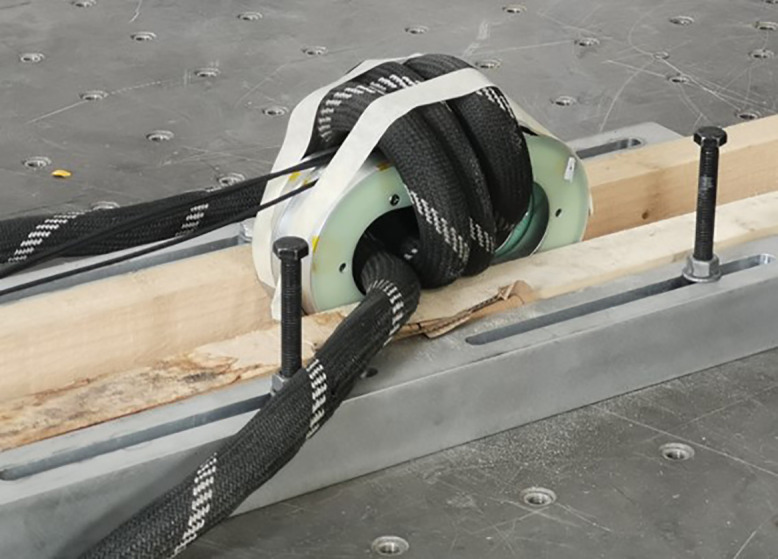
Physical diagram of sensing fiber ring installation.

The sensing fiber ring was fixed on top of the vibration table, as shown in Fig 9. The sensing ring is fixed on the vibration table to ensure that vibration excitation can be applied to the sensing ring. In the sensing ring structure used in this paper, buffer layer is added to the outside of the optical fiber, and anti-impact and anti-pull protective sleeve is added outside the buffer layer to prevent the optical fibers from being squeezed against each other and the optical cable from being subjected to external impact and abnormal pulling.

### 5.2 Experimental results and discussions

**Fig 10:**
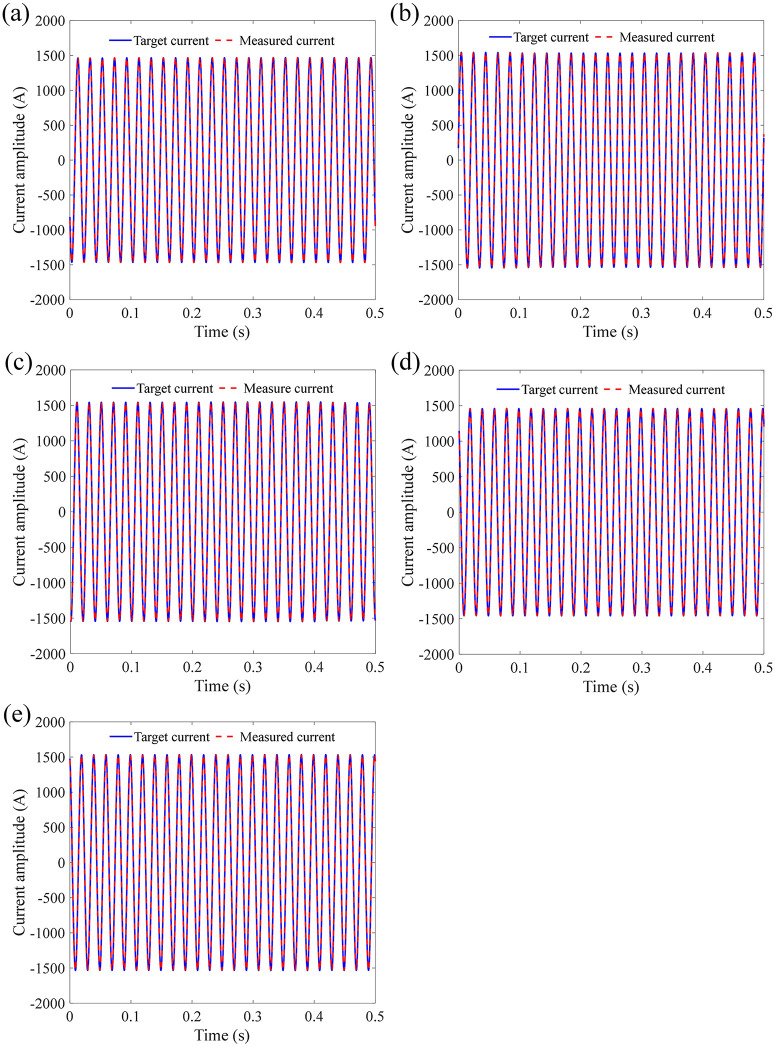
Target and measured current signal under different accelerations and frequencies of sinusoidal vibration: (a) 0.5 g-10 Hz; (b) 0.5 g-150 Hz; (c) 0.5 g-2000 Hz; (d) 2 g-10 Hz; (e) 2 g-2000Hz.

Fig 10 shows the current measurement waveform and the target waveform under different accelerations and frequencies of sinusoidal vibration. It can be seen from Fig 10 that the measured current signal exhibits good coincidence with the target current signal. The RMSE between measured current signal and target current signal is calculated as 36.2745 A, 37.9937 A, 38.0581 A, 35.7818 A, and 37.8034 A, respectively.

**Fig 11:**
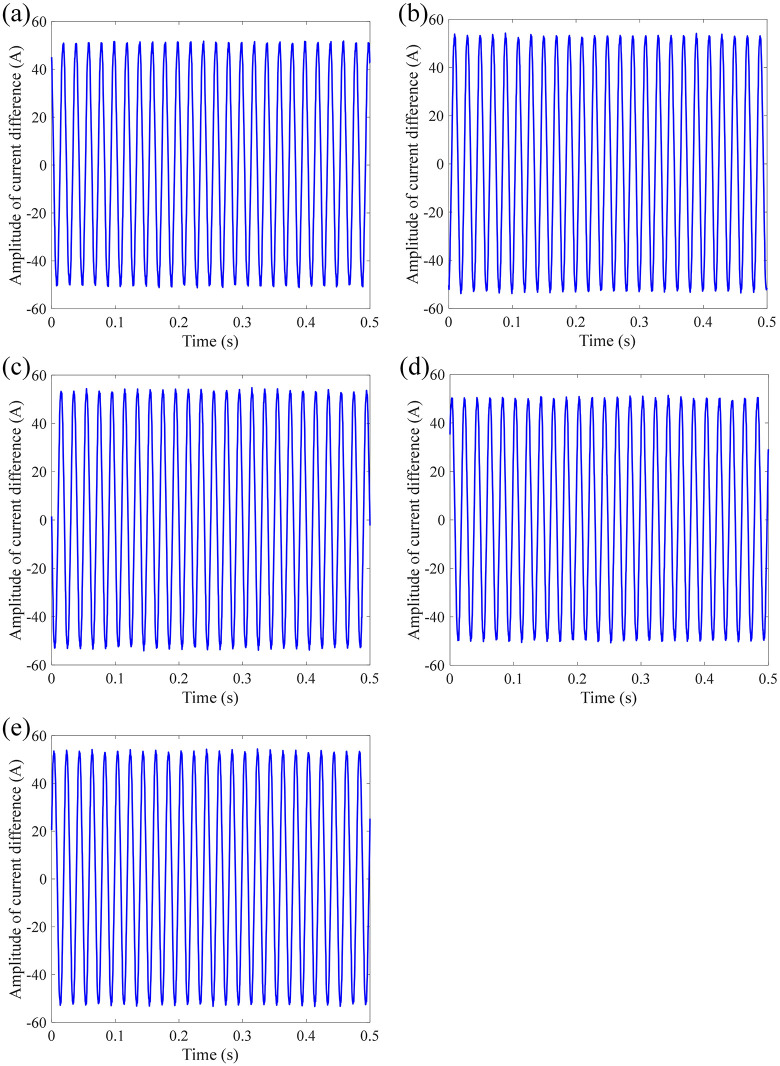
Dividing current signal under different accelerations and frequencies of sinusoidal vibration: (a) 0.5 g-10 Hz; (b) 0.5 g-150 Hz; (c) 0.5 g-2000 Hz; (d) 2 g-10 Hz; (e) 2 g-2000Hz.

Fig 11 shows the dividing signal between the measured current and standard current under different accelerations and frequencies of sinusoidal vibration. The mean absolute error is calculated as 32.6340 A, 34.1228 A, 34.1465 A, 32.1449 A, and 33.9157 A, respectively. Under different influencing factors of sinusoidal vibration of sensing error, the measurement error of FOCT remains within a stable range, proving that the designed anti-vibration structure of the sensing ring is effective for sinusoidal vibration.

**Fig 12:**
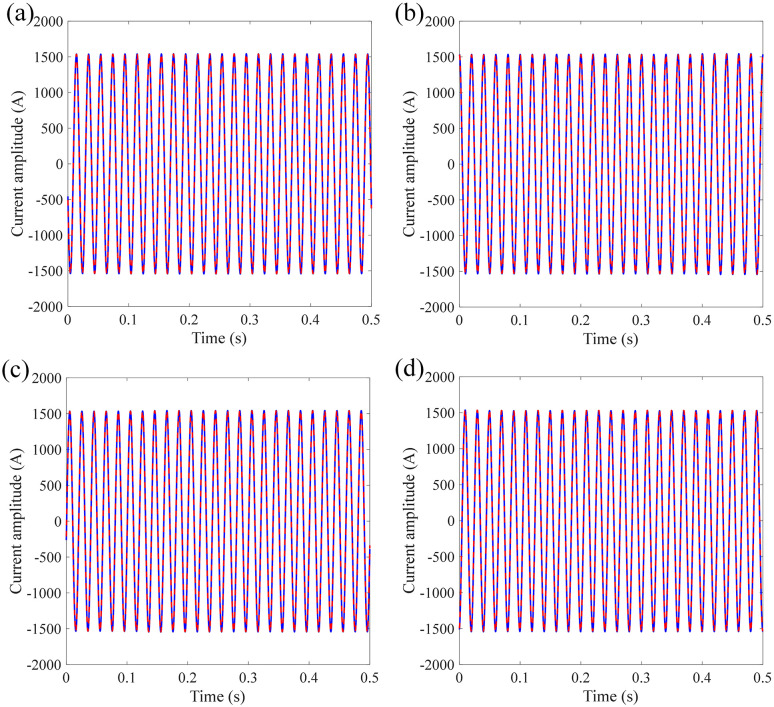
Target and measured current signal under different accelerations and durations of semi-sinusoidal vibration: (a) 5 g-6 ms; (b) 5 g-30 ms; (c) 10 g-6 ms; (d) 10 g-16 ms.

Fig 12 shows the current measurement waveform and the target waveform under different accelerations and durations of semi-sinusoidal vibration. It can be seen from Fig 12 that the measured current signal is in good agreement with the target current signal. The RMSE between measured current signal and target current signal is calculated as 37.8539 A, 37.8 A, 33.9040 A, and 34.3917 A, respectively.

**Fig 13:**
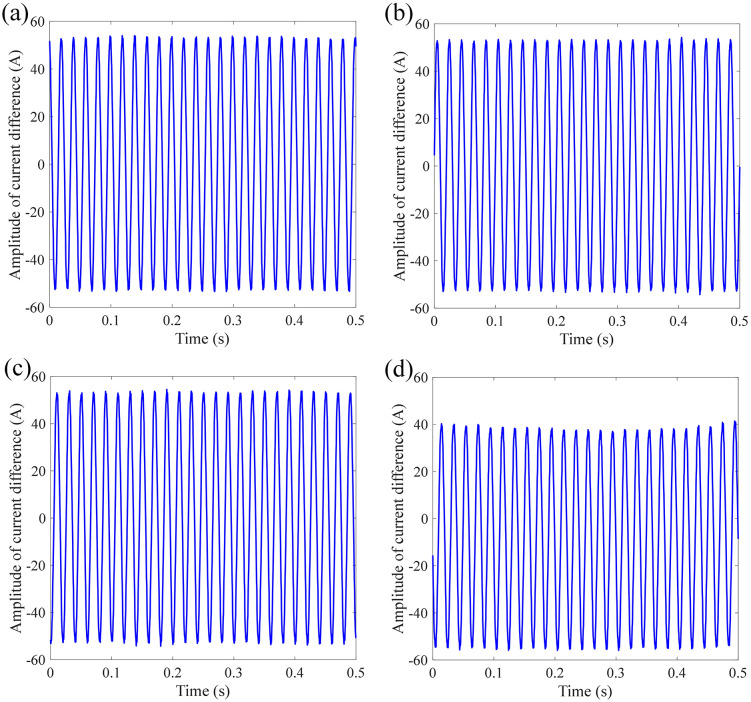
Dividing current signal under different accelerations and durations of semi-sinusoidal vibration: (a) 5 g-6 ms; (b) 5 g-30 ms; (c) 10 g-6 ms; (d) 10 g-16 ms.

Fig 13 shows the dividing signal between the measured current and standard current under different accelerations and durations of semi-sinusoidal vibration. The mean absolute error is calculated as 34.0235 A, 37.9876 A, 34.0897 A, and 30.4802 A, respectively. Under different influencing factors of semi-sinusoidal vibration of sensing error, the measurement error of FOCT remains within a stable range, proving that the designed anti-vibration structure of the sensing ring is effective for semi-sinusoidal vibration.

## 6 Current signal noise suppression method for FOCT under vibration

### 6.1 Vibration noise suppression method of optic fiber of FOCT based on neural network

**Fig 14:**
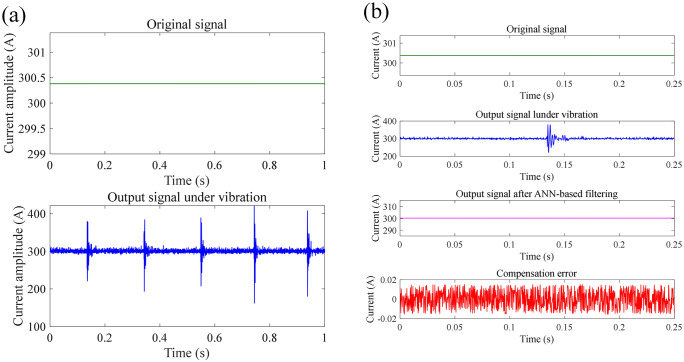
Current measurement compensation results at position 1 under impact vibration: (a) original signal; (b) compensated signal through ANN-based filtering.

**Fig 15:**
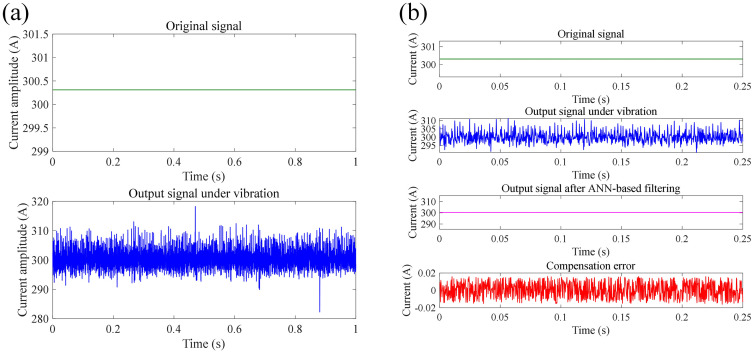
Current measurement compensation results at position 2 under impact vibration: (a) original signal; (b) compensated signal through ANN-based filtering.

**Fig 16:**
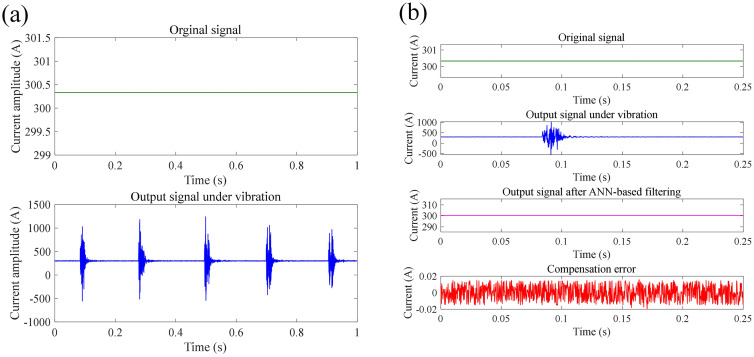
Current measurement compensation results at position 3 under impact vibration: (a) original signal; (b) compensated signal through ANN-based filtering.

In this section, we proposed a neural network-based method to compensate the measurement error of FCOT due to vibration of optic fiber. At each tapping location, 30 sets of signals were measured repeatedly, with a sampling frequency of 4 kHz, and the measurement time for each set of signals was 1 s. The structure of ANN applied for noise filtering of measured current signals of FOCT contains input layer, hidden layer, and output layer. The number of neurons in input layer equals to the number of moments related to the signal value at the current moment. The artificial neural network ANN model includes one hidden layer with 10 neurons. The activation function of ANN model is *Tansig* function in hidden layer, and *Purelin* in output layer. The ANN training function is *Traingd* function, The training rate of the ANN model is 0.1%. The stopping criteria of ANN training is the pre-set learning accuracy. The input dataset for training is the signal fragments containing noise: *x*_noisy_(*t*), *x*_noisy_(*t*-1), ……, *x*_noisy_(*t*-*N* + 1), while the expected output is *x*_clean_(*t*). Training and test dataset contains 2997 and 1000 samples respectively, which all comes from the measured current signal and target current signal shown in Section 4.2. The training of ANN model employs Levenberg-Marquardt method. Figs 14–16 shows the compensation results of measurement error of FOCT under shock vibration at different tapping areas, including the area around modem module, fiber optical ring, and optical cable. It can be seen that the proposed method is able to recover the original current signal from the signal full of noise spikes. The compensation error between original signal and output signal after ANN-based filtering all maintains within −0.02 A to 0.02 A, which is high accuracy for DC current measurement with high amplitude. Quantitatively, target current amplitude given in Figs 14–16 is 300.38 A, 300.31 A, and 300.33 A, respectively. The mean current amplitude after compensation is 300.40 A, 300.33 A, and 300.34 A under shock vibration at three different positions, respectively. Table 1 lists the RMS before and after the compensation through the proposed ANN-based filtering.

**Table 1:** RMS comparison before and after compensation through ANN-based filtering.

Tapping location	Tapping point 1	Tapping point 2	Tapping point 3
RMS before compensation (A)	300.38	300.31	300.33
RMS after compensation (A)	0.0002	0.0154	0.0149

### 6.2 Vibration noise suppression method of sensing ring of FOCT based on FIR filter

In this section, we proposed a FIR filter-based method to recover the original current signal from the measured current signal. The basic mechanism of FIR filter is to perform weighted averaging on the sampled values of the input signal. For a *N*-order FIR filter, its output is determined by the convolution sum of the input *x*[*n*] and the filter coefficients *h*[*k*] (i.e., the unit impulse response):


$$\varphi \left(t\right)=\varphi_{\text{0}}\;sin\left(\omega t\right)$$ (28)

where *y*[*n*] denotes the output at the current moment *n*, *x*[*n* – *k*] denotes the input signals at the current and previous moments (tracing back from the current moment *n* to moments *N* – *n*), *h*[*k*] denotes the kth coefficient of the filter. The output signal after filtering can be expressed as:


$$\varphi \left(t\right)=\varphi_{\text{0}}\;sin\left(\omega t\right)$$ (29)

**Fig 17:**
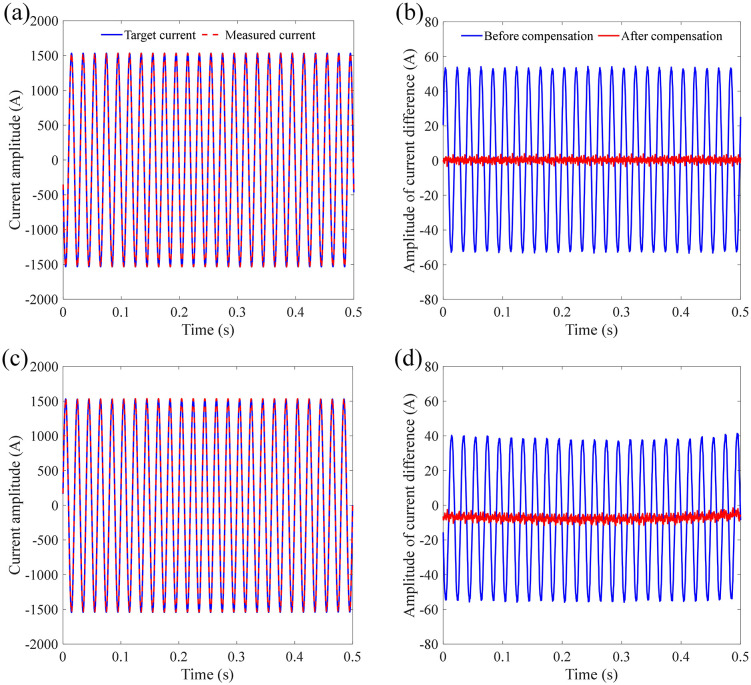
Error compensation results under semi-sinusoidal and sinusoidal vibration based on the proposed FIR filter: (a) measured current signal after compensation (semi-sinusoidal); (b) measurement error after compensation (semi-sinusoidal); (c) measured current signal after compensation (sinusoidal); (d) measurement error after compensation (sinusoidal).

The least squares method is used to solve the optimal filer coefficients. In this work, the order of the FIR filter is 50. Fig 16 shows the current signal after compensation based on the proposed FIR filter along with the comparison of errors before and after compensation. It can be seen from Fig 17 that based on the designed filter, the error between the measurement signal and the target signal has been significantly reduced, whether under semi-sinusoidal vibration or sinusoidal vibration. The absolute error before compensation for the signals shown in Fig 17 is 33.9157 A and 30.4872 A under semi-sinusoidal and sinusoidal vibration, respectively. And the absolute error before compensation for the signals shown in Fig 17 is 1.0124 A and 7.3422 A under semi-sinusoidal and sinusoidal vibration, respectively. The RMSE between measured signal and target signal after compensation is 8.2646 A and 3.5827 A under semi- sinusoidal and sinusoidal vibration, respectively. Table 2 lists the RMS before and after error compensation based on the FIR filtering. The proposed compensation method is effective in reducing RMSE by 75.97% and 90.52% compared with unfiltered data under semi- sinusoidal and sinusoidal vibration, respectively. Hence, it can be concluded that the proposed filter enables effective error compensation to further enhance the measurement accuracy of FOCT under the vibration of the sensing ring.

**Table 2:** RMS comparison before and after compensation based on FIR filtering.

Vibration type	Semi-sinusoidal vibration	Sinusoidal vibration
RMS before compensation (A)	8.2646	3.5827
RMS after compensation (A)	34.3917	37.8034

## 7 Conclusions

This research provided a detailed analysis of the impact of vibration on Fiber Optic Current Transformers (FOCTs), offering insights into the specific components most vulnerable to vibrational interference. By clarifying the mechanisms through which vibration affects components such as polarization-maintaining delay fiber and sensing fiber, this study laid the groundwork for targeted mitigation strategies. The experimental investigations under various vibration conditions allowed for a thorough characterization of FOCT performance, revealing the nuanced effects of different vibration types on measurement accuracy.

The proposed solutions, centered around a novel sensing ring design and adaptive signal processing, demonstrated significant potential in enhancing FOCT robustness. The implementation of ANN-based and FIR filtering-based methods successfully compensated for measurement errors induced by both sinusoidal and impact vibrations, indicating the effectiveness of these techniques in real-time error correction. These findings highlight the potential of intelligent signal processing in improving the reliability of FOCTs in challenging operational environments.

Ultimately, this work contributes to the advancement of FOCT technology by addressing a critical challenge: vibration-induced measurement inaccuracies. The combined approach of innovative structural design and adaptive signal processing offers a promising path towards enhancing the operational stability and reliability of FOCTs in diverse applications. By providing a solid theoretical foundation and practical solutions, this research paves the way for wider adoption of FOCTs in demanding environments where accurate and dependable current measurement is essential. The outcomes of this study not only benefit the immediate field of fiber optic current sensing but also have broader implications for other optical sensing technologies facing similar challenges in vibration-prone settings.

## Supporting information

S1 Data Data used in this manuscript. (ZIP)

## References

[1] Xu S-Y, Xing F-F, Li W, Wang Y-Q, Wang X-H, Wang R-L. A Stray Current Sensor Based on an All-Side Cylindrical Spiral Fiber. IEEE Photonics J. 2017;9(1):1–14. doi: 10.1109/jphot.2016.2646365

[2] Ochoa M, Algorri JF, Roldán-Varona P, Rodríguez-Cobo L, López-Higuera JM. Recent Advances in Biomedical Photonic Sensors: A Focus on Optical-Fibre-Based Sensing. Sensors (Basel). 2021;21(19):6469. doi: 10.3390/s21196469 34640788 PMC8513032

[3] Naeem ZJ, Salman AM, Faris RA, Al-Janabi A. Highly efficient optical fiber sensor for instantaneous measurement of elevated temperature in dental hard tissues irradiated with an Nd:YaG laser. Appl Opt. 2021;60(21):6189–98. doi: 10.1364/AO.431369 34613285

[4] Sima W, Zeng L, Yang M, Yuan T, Sun P. Improving the Temperature and Vibration Robustness of Fiber Optic Current Transformer Using Fiber Polarization Rotator. IEEE Trans Instrum Meas. 2022;71:1–12. doi: 10.1109/tim.2021.3139700

[5] Noori NF, Mansour TS. A review of recently optical hybrid optical fiber interferometers. J Opt. 2025;54(5):3698–711. doi: 10.1007/s12596-025-02588-9

[6] Bae G, Kim J, Choi S, Bae M, Wook Lee Y. Temperature-Robust All-Fiber Demodulation of Optical Current Sensor Using Long-Period Grating on Birefringent Photonic Crystal Fiber. IEEE Sensors J. 2024;24(22):36854–62. doi: 10.1109/jsen.2024.3467226

[7] Xiang L, Pang F, Xiao Z, Zhang L, Wei H, Zhu M, et al. Vibration-insensitive polarimetric fiber optic current sensor based on orbital angular momentum modes in an air-core optical fiber. Opt Lett. 2024;49(7):1753–6. doi: 10.1364/OL.519974 38560854

[8] Yu A, Li C, Huang J, Tian J, Xiang P, Ke Y, et al. Temperature and Vibration Resistant Fiber-Optic Current Sensor With Reflective Recirculating Loop. J Lightwave Technol. 2025;43(16):7932–8. doi: 10.1109/jlt.2025.3576263

[9] Descamps F, Aerssens M, Gusarov A, Mégret P, Massaut V, Wuilpart M. Simulation of vibration-induced effect on plasma current measurement using a fiber optic current sensor. Opt Express. 2014;22(12):14666–80. doi: 10.1364/OE.22.014666 24977562

[10] Rogers AJ, Jincheng Xu, Jialing Yao. Vibration immunity for optical-fiber current measurement. J Lightwave Technol. 1995;13(7):1371–7. doi: 10.1109/50.400674

[11] Short SX, Tantaswadi P, de Carvalho RT, Russell BD, Blake J. An experimental study of acoustic vibration effects in optical fiber current sensors. IEEE Trans Power Delivery. 1996;11(4):1702–6. doi: 10.1109/61.544246

[12] Bohnert K, Gabus P, Nehring J, Brandle H. Temperature and vibration insensitive fiber-optic current sensor. J Lightwave Technol. 2002;20(2):267–76. doi: 10.1109/50.983241

[13] Jiang ZY, Zhang CX, Feng LS, Wang XX. Study of temperature character on fiber-optical current transducer. In: OFCIO, 2005. 787–92.

[14] Wang XX, Zhang CX, Jiang ZY, Yuan YC. Optimize the design of the quarter-waveplate in all fiber optical transformers. Optic Techn. 2005;31:241–3.

[15] Yuan YC, Feng LS, Wang XX, Jiang ZY. Design of phase difference measurement system on all-fiber optical current transformers. In: The 12th National Conference on Fiber Optic Communication and the 13th Academic Conference on Integrated Optics. 2005. p. 923–6.

[16] Zhao JJ, Feng LS, Zhang CX. Optimum design of fiber optical current sensor. Optic Techn. 2005;31(6):119–21.

[17] Mu J, Wang J, Zhao W, Xu JT. Vibration and temperature insensitive fiber-optic current sensor. High Voltage Eng. 2010;36(4):980–6.

[18] Li XY, Hao JH, Yang HR, Yang JW, Chen L, He Z. Research on the Compensating Fiber Loop for Eliminating Vibration in Sagnac Optic Current Sensor. Chin J Laser. 2012;39(2):0205005. doi: 10.3788/cjl201239.0205005

[19] Kang M, Wang Y, Xu J, Ren K, Liu S. Vibration immunity fiber optic current sensor employing a spun or twisted highly linear birefringence fiber. In: Proceedings of the 32nd Chinese Control Conference. 2013. p. 7468–72.

[20] Wang YL, Kang MH, Ren LY, Ren KL. Design of spun high-birefringent fiber for fiber optic current sensor. Infrared Laser Eng. 2015;44(1):170–5.

[21] Kim S-M, Dandu P, Gusarov A, Danisi A, Vayakis G, Wuilpart M. Assessment of the Structural Vibration Effect on Plasma Current Measurement Using a Fiber Optic Current Sensor in ITER. Sensors (Basel). 2023;23(3):1460. doi: 10.3390/s23031460 36772496 PMC9921135

[22] Xiang L, Pang F, Xiao Z, Zhang L, Wei H, Zhu M, et al. Vibration-insensitive polarimetric fiber optic current sensor based on orbital angular momentum modes in an air-core optical fiber. Opt Lett. 2024;49(7):1753–6. doi: 10.1364/OL.519974 38560854

[23] Sima W, Zeng L, Yang M, Yuan T, Sun P. Improving the Temperature and Vibration Robustness of Fiber Optic Current Transformer Using Fiber Polarization Rotator. IEEE Trans Instrum Meas. 2022;71:1–12. doi: 10.1109/tim.2021.3139700

[24] Zheng S, Ren M, Luo X, Zhang H, Feng G. Real-Time Compensation for SLD Light-Power Fluctuation in an Interferometric Fiber-Optic Gyroscope. Sensors (Basel). 2023;23(4):1925. doi: 10.3390/s23041925 36850522 PMC9965770

[25] Kim S-M, Dandu P, Gusarov A, Danisi A, Vayakis G, Wuilpart M. Assessment of the Structural Vibration Effect on Plasma Current Measurement Using a Fiber Optic Current Sensor in ITER. Sensors (Basel). 2023;23(3):1460. doi: 10.3390/s23031460 36772496 PMC9921135

[26] Wu J, Zhang X, Chen L. Research on the Dual Modulation of All-Fiber Optic Current Sensor. Sensors (Basel). 2022;22(2):430. doi: 10.3390/s22020430 35062391 PMC8781253

[27] Zhang Z, Zhang X, Li B, Qin S, Ding L. Temperature characterization of fiber optic current sensor influenced by polarization-maintaining transmission fiber. Sens Actuators A. 2024;377:115779. doi: 10.1016/j.sna.2024.115779

[28] Hao Z, Pu S, Lahoubi M, Zhang C, Liu W. Dual-channel-in-one temperature-compensated all-fiber-optic vector magnetic field sensor based on surface plasmon resonance. Opt Express. 2023;31(3):4826–38. doi: 10.1364/OE.481161 36785440

[29] Jiang Z, Zhang L, Wang G, Jin L. Sensitivity and Noise Analysis of Resonant All-Fiber Optic Current Transformers. IEEE Photonics J. 2024;16(4):1–8. doi: 10.1109/jphot.2024.3412237

[30] Yao H, Hu G, Wang X. Design of Amplifier Circuit of Optical Fiber Sensor and Its Application in Cloud Computing of Mechanical Vibration Fault. J Nanoelectron Optoelectron. 2023;18(2):202–9. doi: 10.1166/jno.2023.3378

[31] Lopez JD, Dante A, Bacurau RM, Cremonezi AO, Mok RW, Carvalho CC, et al. Fiber-Optic Current Sensor Based on FBG and Optimized Magnetostrictive Composite. IEEE Photon Technol Lett. 2019;31(24):1987–90. doi: 10.1109/lpt.2019.2952255

[32] Liu Y, Xiong J, Huang J, Pang F, Zhao Y, Xia L. Passive Time-Division Multiplexing Fiber Optic Sensor for Magnetic Field Detection Applications in Current Introduction. Photonics. 2025;12(5):506. doi: 10.3390/photonics12050506

[33] Zhang SZ, Liu QW, Liu ST, He ZY. Closed-Loop Resonant Fiber Optic Current Sensor Based on Broadband Source and Linear Cavity. J Lightwave Technol. 2025;43(17):8452–9.

[34] Li J, Li S, Chen H, Yin Z, Shao P, Li K, et al. High-Sensitivity Twist Sensor Based on Sagnac Interference With a Helical Elliptical Core Fiber. IEEE Sensors J. 2024;24(9):14335–42. doi: 10.1109/jsen.2024.3380832

[35] Li L, Lv D, Yang M, Xiong L, Luo J. A IR-Femtosecond Laser Hybrid Sensor to Measure the Thermal Expansion and Thermo-Optical Coefficient of Silica-Based FBG at High Temperatures. Sensors (Basel). 2018;18(2):359. doi: 10.3390/s18020359 29373528 PMC5856160

[36] Huang YH, Xia L, Pang FB, Yuan YB, Ji JF. Self-Compensative Fiber Optic Current Sensor. J Lightwave Technol. 2021;39(7):2187–93.

[37] Zhu X, Zhao X, Liu X, Ren Z. Temperature stability analysis of the all-fiber current sensor with a loop structure. Laser Phys. 2024;34(12):125103. doi: 10.1088/1555-6611/ad8cb6

[38] Xu S, Li W, Xing F, Wang Y, Wang R, Wang X. An Elimination Method of Temperature-Induced Linear Birefringence in a Stray Current Sensor. Sensors (Basel). 2017;17(3):551. doi: 10.3390/s17030551 28282953 PMC5375837

[39] Izdebski M, Ledzion R, Kucharczyk W. Precise Method for Measuring the Quadratic Electro-Optic Effect in Noncentrosymmetric Crystals in the Presence of Natural Birefringence. Materials (Basel). 2020;13(18):3942. doi: 10.3390/ma13183942 32899980 PMC7559402

