# Correction: Anti-vibration method for sensing ring of fiber optical current transformer integrating structural design and adaptive signal processing

**DOI:** 10.1371/journal.pone.0352942

**Published:** 2026-07-06

**Authors:** The PLOS One Editors

## Notice of Republication

This article [1] was republished on April 21, 2026, to address an issue identified post-publication. Please download this article again to view the correct version.

Additionally, the funding statement is updated to: This work is financially supported by the Scientific and Technological Project of State Grid Corporation of China (program 5700-202332628A-3-2-ZN to L.C.).

Furthermore, a duplicate of this article was erroneously published under a different DOI on February 10, 2026 [2, retracted in 3].

The duplicate has since been retracted by the publisher; this does not impact the standing of [1].

## References

[1] Cheng L, Luo J, Si W, Han Y, Zuo K, Sun H, et al. Anti-vibration method for sensing ring of fiber optical current transformer integrating structural design and adaptive signal processing. PLoS One. 2026;21(2):e0341890. doi: 10.1371/journal.pone.0341890 41758877 PMC12948120

[2] Cheng L, Luo J, Si W, Han Y, Zuo K, Sun H, et al. RETRACTED: Anti-vibration method for sensing ring of fiber optical current transformer integrating structural design and adaptive signal processing. PLoS One. 2026;21(2):e0342161. doi: 10.1371/journal.pone.0342161 41666227 PMC12890127

[3] The *PLOS One* Editors. Retraction: Anti-vibration method for sensing ring of fiber optical current transformer integrating structural design and adaptive signal processing. PLoS One. 2026;21(7):e0352943. doi: 10.1371/journal.pone.0352943 PMC13336200 42406703

